# Structure of the Kaposi’s sarcoma-associated herpesvirus gB in post-fusion conformation

**DOI:** 10.1128/jvi.01533-24

**Published:** 2025-01-17

**Authors:** Fumiaki Ito, James Zhen, Guodong Xie, Haigen Huang, Juan C. Silva, Ting-Ting Wu, Z. Hong Zhou

**Affiliations:** 1 Department of Microbiology, Immunology, and Molecular Genetics, University of California, Los Angeles (UCLA) 8783 , Los Angeles, California, USA; 2 California NanoSystems Institute, UCLA 8783 , Los Angeles, California, USA; 3 Molecular Biology Institute, UCLA 8783 , Los Angeles, California, USA; 4 Department of Molecular and Medical Pharmacology, David Geffen School of Medicine, UCLA 8783 , Los Angeles, California, USA; Lerner Research Institute, Cleveland Clinic, Cleveland, Ohio, USA

**Keywords:** Kaposi's sarcoma-associated herpesvirus, glycoproteins, structural biology, electron microscopy

## Abstract

Discovered in 1994 in lesions of an AIDS patient, Kaposi’s sarcoma-associated herpesvirus (KSHV) is a member of the gammaherpesvirus subfamily of the *Herpesviridae* family, which contains a total of nine that infect humans. These viruses all contain a large envelope glycoprotein, glycoprotein B (gB), that is required for viral fusion with host cell membrane to initial infection. Although the atomic structures of five other human herpesviruses in their postfusion conformation and one in its prefusion conformation are known, the atomic structure of KSHV gB has not been reported. Here, we report the first structure of the KSHV gB ectodomain determined by single-particle cryogenic electron microscopy (cryoEM). Despite a similar global fold between herpesvirus gB, KSHV gB possesses local differences not shared by its relatives in other herpesviruses. The glycosylation sites of gB are arranged in belts down the symmetry axis with distinct localization compared to that of other herpesviruses, which occludes certain antibody binding sites. An extended glycan chain observed in domain I (DI), located proximal to the host membrane, may suggest its possible role in host cell attachment. Local flexibility of domain IV (DIV) governed by molecular hinges at its interdomain junctions identifies a means for enabling conformational change. A mutation in the domain III (DIII) central helix disrupts incorporation of gB into KSHV virions despite adoption of a canonical fold *in vitro*. Taken together, this study reveals mechanisms of structural variability of herpesvirus fusion protein gB and informs its folding and immunogenicity.

**IMPORTANCE:**

In 1994, a cancer-causing virus was discovered in lesions of AIDS patients, which was later named Kaposi’s sarcoma-associated herpesvirus (KSHV). As the latest discovered human herpesvirus, KSHV has been classified into the gammaherpesvirus subfamily of the *Herpesviridae*. In this study, we have expressed KSHV gB and employed cryogenic electron microscopy (cryoEM) to determine its first structure. Importantly, our structure resolves some glycans beyond the first sugar moiety. These glycans are arranged in a pattern unique to KSHV, which impacts the antigenicity of KSHV gB. Our structure also reveals conformational flexibility caused by molecular hinges between domains that provide clues into the mechanism behind the drastic change between prefusion and postfusion states.

## INTRODUCTION

Kaposi’s sarcoma-associated herpesvirus (KSHV), the most recently identified human herpesvirus, was discovered in the lesions of acquired immunodeficiency syndrome (AIDS) patients (1). KSHV is a member of the gammaherpesvirus subfamily and is the causative agent of several devastating diseases, including Kaposi’s sarcoma, primary effusion lymphoma, and multicentric Castleman’s disease (2, 3). In particular, KSHV imposes a cancer burden on immunosuppressed individuals, such as AIDS patients and organ transplant recipients (4). Despite the potential for severe complications, there are currently no vaccines or treatments against KSHV infections to prevent its associated malignancies (5, 6).

Like all herpesviruses, KSHV is composed of a dsDNA genome within a capsid that is further surrounded by a tegument layer and enclosed inside of an envelope decorated with glycoproteins. These glycoproteins play critical roles in mediating membrane fusion during both the maturation and infection processes (7–9). After nuclear egress of the capsid into the cytosol, the tegumented capsid acquires two sets of envelopes during the secondary envelopment process. The outer envelope layer fuses with the host membrane from the cytosolic side to release the now single-enveloped mature virion (9). During host entry, the viral envelope attaches to and fuses with the host membrane to allow entry of the genome-containing capsid (10). Because of the critical nature of these membrane fusion events for the life cycle of KSHV, its envelope glycoproteins responsible for membrane fusion are prime targets for vaccine and therapeutic development.

Glycoprotein B (gB) is the fusogen responsible for the physical action of membrane fusion and is conserved across all herpesviruses. As a class III fusion protein (11, 12), gB undergoes a conformational change from a prefusion state to a postfusion state that draws the host and viral envelopes together to induce fusion. Structural determination of gB in the prefusion and postfusion conformations would provide insights into the herpesvirus fusion mechanism and its epitopes. Structures of postfusion gB have previously been determined for herpes simplex virus type 1 (HSV-1), Epstein-Barr virus (EBV), human cytomegalovirus (HCMV), and varicella-zoster virus (VZV) by crystallography (13–20). However, the structure of gB in the prefusion state has been elusive due to its metastability. Cryogenic electron tomography (cryoET) has been used to observe gB in the context of intact membranes and subsequently identify its prefusion conformation (21–25). Recently, single -particle cryogenic electron microscopy (cryoEM) has been used to determine the prefusion structures of HCMV gB at near-atomic resolution (26, 27).

Despite these advancements in the structural determination of gB, the structure of KSHV gB in neither postfusion state nor prefusion state has been determined. In addition to a lack of KSHV gB structures, the effects of glycosylation on KSHV gB are not well understood. Viruses employ glycosylation to shield its potential neutralizing epitopes from recognition by host antibodies (28, 29). Each herpesvirus gB possesses varying degrees of glycosylation at different sites, which should impact the accessibility of antibody binding sites. Structures of KSHV gB in tandem with the gB structures of its fellow herpesviruses would enable a more holistic understanding of their fusion mechanism and immunogenicity.

In this study, we determined the structure of KSHV gB in its postfusion conformation by cryoEM. In our structure, we observe glycans beyond the first sugar moiety. The glycosylation sites of KSHV gB alternate between belts of glycan-rich and glycan-poor surfaces around the ectodomain, which dictate the availability of epitope sites. Additionally, KSHV gB contains unique, local structural features distinct from the conserved folds of gB in other herpesviruses. We further observed that the domain IV (DIV) crown of KSHV gB experiences rotational flexibility governed by hinges at its interdomain junctions.

## RESULTS

### Overall structure of KSHV gB

**Fig 1:**
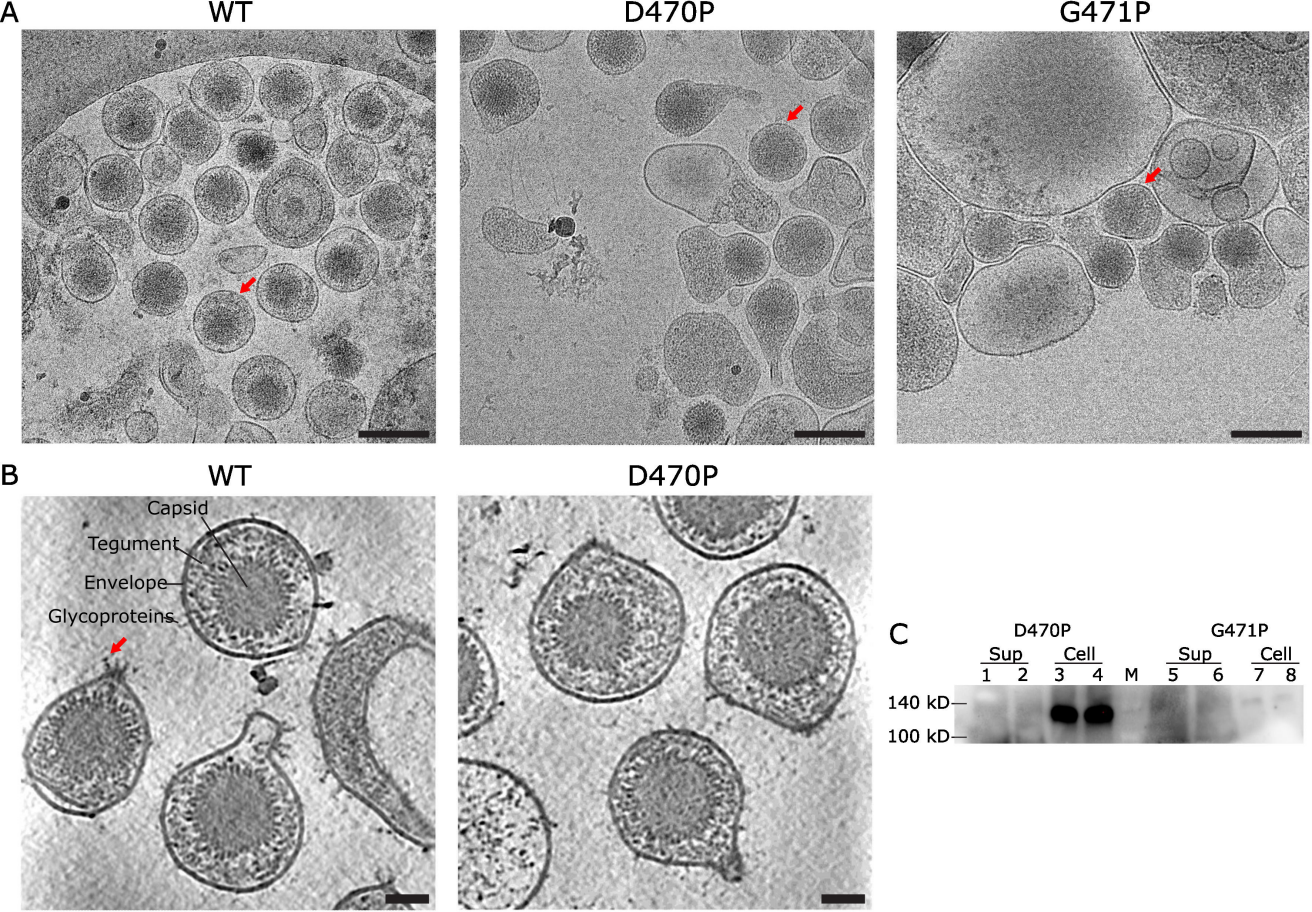
KSHV virions. (**A**) CryoEM images of KSHV virions expressing WT gB (left), gB D470P (middle), and gB G471P (right). The red arrow indicates example mature virion. Scale bar, 200 nm. (**B**) Tomogram of KSHV expressing WT gB (left) and gB D470P (right). The red arrow indicates example postfusion gB. Scale bar, 50 nm. (**C**) Western blot for expression of the gB mutant in iSLK-D470P or iSLK-G471P cells. Lanes 1, 2, 5, and 6: media; Lanes 3, 4, 7, and 8: cell lysates. Lanes 1–4: iSLK-D470P and Lanes 5–8: iSLK-G471P. Lanes 1, 3, 5, and 7: 1 mM NaB plus 5 µg/ml tetracycline. Lanes 2, 4, 6 and 8: 1 mM NaB plus 7.5 µg/ml tetracycline. M: protein ladder. The membrane was probed with an anti-FLAG antibody.

To understand the mechanisms of membrane fusion and cell entry mediated by KSHV, we introduced mutations in gB in an attempt to enrich for its prefusion state on otherwise native KSHV virions. Prior cryoET studies of gB from HSV-1 and VZV demonstrated that a single proline mutation within ^515^RHV^517^ in HSV-1 or ^526^EHV^528^ in VZV near the DIII hinge region, was sufficient to stabilize its prefusion conformation on extracellular vesicles. We selected equivalent residues, ^470^DGI^472^, in KSHV to test if proline substitutions at this location could retain the prefusion conformation (Fig. S1A) (22, 23). Both D470P and G471P mutant KSHVs were able to produce mature virions, although G471P mutant had a lower yield than D470P (Fig. 1A and B). Through immunoblot analysis, D470P gB is not detected in the supernatant from which secreted virions were isolated or virions concentrated from the supernatant but is detected in the cellular lysates (Fig. 1C; Fig. S1B). On closer inspection through cryoET, KSHV gB D470P mutant virions do not have distinct densities of postfusion gB like those on KSHV WT virions (Fig. 1B).

**Fig 2:**
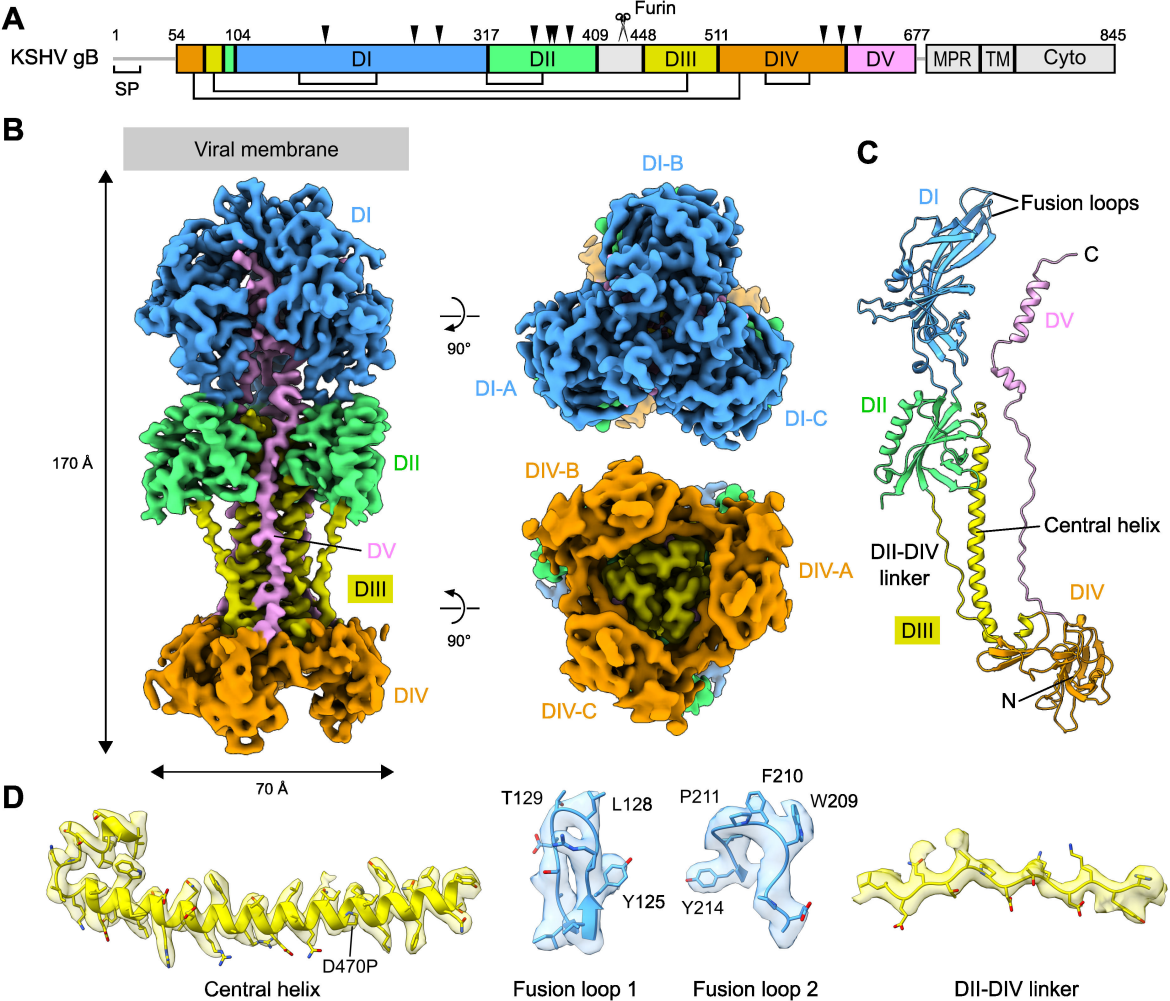
Structure of KSHV gB ectodomain. (**A**) Schematic representation of the domain organization and construct design of KSHV gB. SP, signal peptide; MPR, membrane proximal region; TM, transmembrane; Cyto, cytoplasmic region. The regions either invisible in the cryoEM map or excluded from the construct are colored in light gray. N-linked glycosylation sites observed in the structure are indicated with black triangles above the schematic boxes. Five conserved disulfide bonds are depicted with black lines below the boxes. (**B**) Three orthogonal views of the cryoEM map of the KSHV gB ectodomain trimer colored by the five core domains. (**C**) Atomic model of a protomer of the gB ectodomain trimer colored by the five core domains. (**D**) Exemplary cryoEM densities highlighting the segments of DI and DIII. The densities are shown in semi-transparent surfaces superimposed with their corresponding atomic models in ribbons and sticks.

To gain more insights into structural basis of KSHV gB, we expressed and purified KSHV gB containing these mutations for structural determination by cryoEM. We initially tried to use full-length constructs but encountered problems with sample preparation of this membrane protein. To alleviate this issue, we truncated the sequence and determined the structure of the soluble ectodomain of KSHV gB (amino acid residues 1–687). The ectodomain construct includes five subdomains DI–DV but excludes the C-terminal membrane proximal region (MPR), transmembrane domain (TM), and cytoplasmic domain (Fig. 2A). Of the homologous mutation sites at the DIII central helix (22, 23), D470P was selected for structural determination for its expression level and charged sidechain (Fig. S1C). The gB ectodomain was expressed in and purified from mammalian cells in its fully glycosylated form.

The structure of KSHV gB was determined at 3.3 Å resolution by single particle cryoEM (Fig. S2 and S3; Table S1). The gB structure was reconstructed as a homotrimer with C3 symmetry aligned along the long edge. KSHV gB ectodomain exhibits an elongated rod-shaped architecture with dimensions approximately 70 Å by 70 Å by 170 Å, consistent with the postfusion conformation observed in other herpesvirus fusion proteins, such as those from HSV-1, VZV, HCMV, and EBV (13–15, 20) (Fig. 2B). We did not observe any 2D or 3D classes representing potential prefusion form (Fig. S2), which is predicted to have a more globular dimension, highlighting the metastable nature of the prefusion conformation. Additionally, no asymmetric assembly was observed from 3D refinement without imposing symmetry, and further 3D classification did not yield any asymmetric states, indicating minimal conformational variability among the three individual protomers.

The cryoEM map resolved the densities for residues 54–677 consisting of the five core domains (Fig. 2C). The KSHV gB follows a canonical postfusion domain organization of the class III fusion protein, with its core comprising three β-strand-rich modular domains (DI, DII, and DIV) and a long three-helix bundle of DIII. DI forms a trimer at the membrane-proximal end of the ectodomain with its hydrophobic fusion loops facing the membrane side. DII is juxtaposed to DI through two linkers and encircles the N-terminal end of the DIII central helical bundle. DIII bridges DII and DIV via the central helices and a DII-DIV hanging linker. DIV trimer is positioned at the membrane-distal end of the ectodomain. Finally, DV folds back and extends from DIV to DI along the DIII central helices, with its two C-terminal helices intercalating into the DI trimer interface. The C-terminal end of the DV exits between the DI-DI interface potentially allowing the subsequent MPR/TM region to form a trimeric bundle atop the DI trimer (Fig. 2A).

Other than these resolved regions, the first 53 residues at the N-terminus, containing signal peptide (residues 1-23) followed by a region rich in Pro, Ser, and Thr residues, had no density, indicating that this region is disordered. The residues 409-447 that link DII and DIII, including the furin cleavage site (^437^RKRR/S^441^) also lack density in the cryoEM map. This region is highly variable in both sequence and length among herpesvirus gB proteins. A 14-residue patch area preceding the furin cleavage site (^419^PTSSPPPSASPMTTS^433^) that is also rich in Pro, Ser, and Thr could potentially be modified with O-linked glycosylation as previously shown for HSV-1 gB (30, 31). The flexibility and proteolytic cleavage in this segment are likely important for the pre- to postfusion conformational transition as the spatial orientation between DII and DIII is inverted by nearly 180 degrees during the transition (26). The five conserved disulfide bonds were readily observed, including four intra-domain bonds in DI (C157-C222), DIII (C85-C484), and DIV (C68-C528 and C550-C587), and one that crosslinks between the DI-DII linker and DII (C315-C362), providing rigidity to the DI-DII junction (Fig. S4).

The cryoEM map of the KSHV gB displays amino acid side chain densities for most regions (Fig. 2D; Fig. S5). The density of the DIII central helix is shown in Fig. 2D. The two fusion loops in DI that are located at the edge of the ectodomain were previously missing in the cryoEM structure of closely related EBV gB (PDB ID: 7FBI) (32). Our map has well-resolved density for these regions, which allowed us to build the corresponding atomic models. Additionally, a continuous density for the DII-DIV hanging linker region of DIII, previously unresolved in EBV gB, was observed with a moderate resolution that permitted model building (Fig. 2D).

### Unique glycosylation pattern of KSHV gB

**Fig 3:**
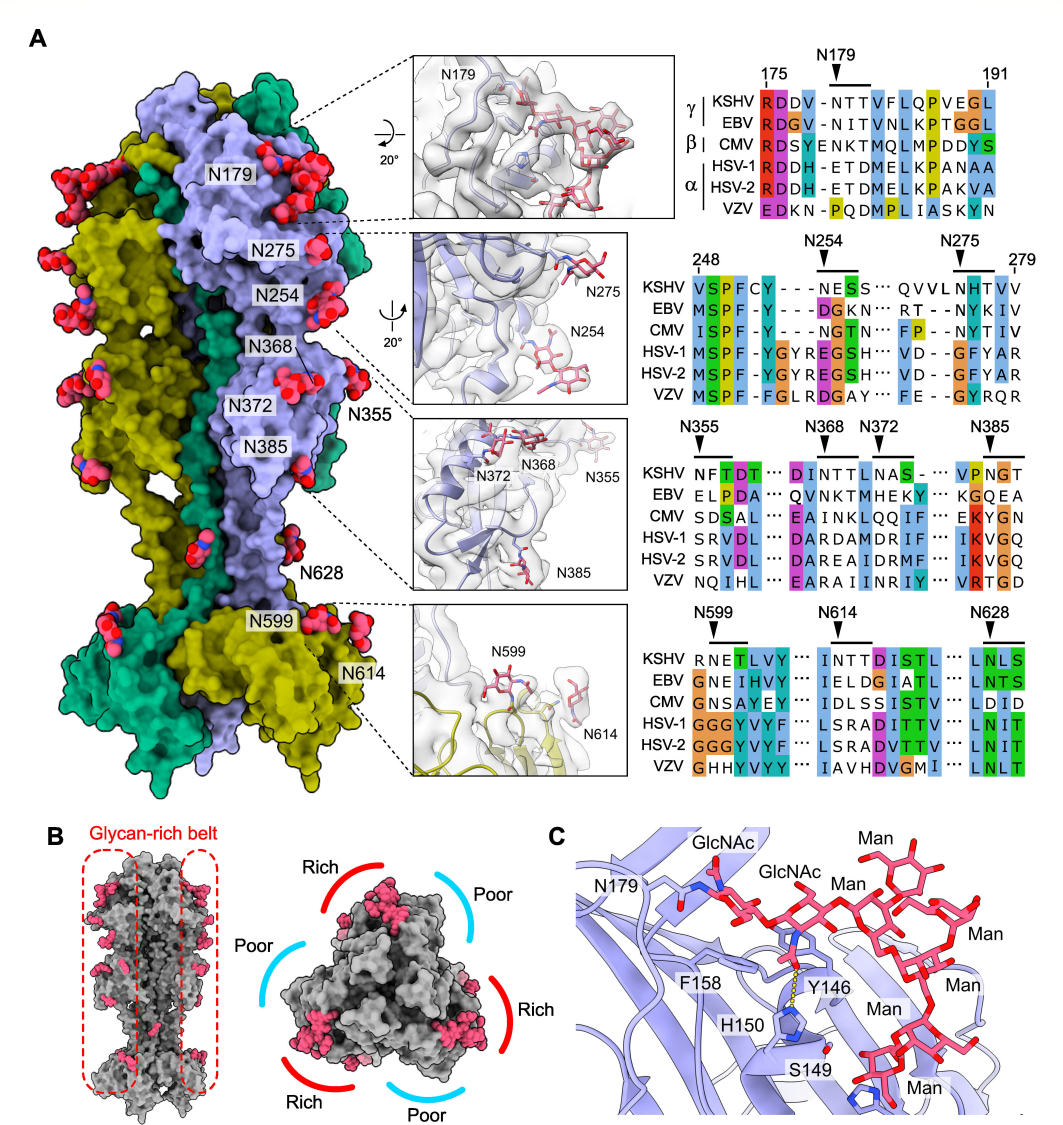
Glycan chain of KSHV gB. (**A**) Overview of the surface glycosylation of the KSHV gB ectodomain trimer. The atomic models of the proteins are shown in surface representation, and the glycan chains are shown in spheres. The close-up views of the glycosylation sites are shown in the insets in the center. The corresponding cryoEM densities are superimposed on the atomic models. Amino acid sequence alignments near the glycosylation sites are shown on the right to highlight the conservation of the glycosylation sites. (**B**) Glycan chain distribution pattern on the KSHV gB surface viewed from the side (left) and top (right), highlighting the glycan-rich and glycan-poor surface areas. (**C**) Close-up view of the glycan chain at N179. High-mannose glycan chain has been modeled based on the observed cryoEM density. The first two GlcNAcs form stacking interactions with the aromatic side chains of Phe158 and Tyr146, respectively.

Herpesvirus gB proteins are extensively glycosylated to promote the evasion of neutralizing antibody recognition and host cell attachment (33). Besides amino acid polypeptide densities, we observed extra densities distributed across the gB surface that connect to the side chain densities of the surface-exposed asparagine residues. These asparagine residues are part of a sequence motif of Asn-X-(Ser/Thr), where X represents any amino acid except proline, characteristic of N-linked glycosylation. Therefore, we attributed these extra densities to N-linked glycans. Of the 13 hypothetical glycosylation sites per protomer based on consensus sequence in KSHV gB, 10 sites displayed the densities for glycan chains. They are distributed across domains DI, DII, DIV, and DV at asparagine residues 179, 254, 275, 355, 368, 372, 385, 599, 614, and 628 (Fig. 3A). We could not observe glycosylation at the hypothetical sites at residues 408, 455, and 562. Asn408 and 562 are located in regions of relatively weak cryoEM density near the unmodeled DII-DIII junction and DIV, respectively. Asn455, located at the N-terminal base of the DIII central helix, is engaged in a delocalized π-electron network that stabilizes the coiled-coil and sterically occludes glycosylation. No O-linked glycosylation was observed in the resolved region of the cryoEM density map. Notably, the glycosylation sites on the gB surface are predominantly clustered along a belt that extends along the symmetry axis of the gB trimer from DI to DIV (Fig. 3B). This surface glycosylation distribution pattern creates glycan-rich and glycan-poor surface areas that alternate along the symmetry axis and potentially influences the antigenicity of the gB surface.

Most glycan chains displayed discernible density only for the first one or two sugar moieties, specifically N-acetylglucosamine (GlcNAc), due to the intrinsic flexibility of the sugar chains. Notably, the glycan at N179 showed an extended sugar chain density, corresponding to up to eight sugar molecules, including two branching points (Fig. 3C). At this site, the initial two GlcNAcs form stacking interactions with the aromatic side chains of Phe158 and Tyr146, respectively. The second GlcNAc forms a hydrogen bond through the oxygen atom of its N-acetyl group with the nitrogen atom of the side chain imidazole ring of His150, thereby stabilizing the glycan chain. The observed branching pattern and sugar chain architecture indicate that this glycan chain belongs to the oligomannose type among the potential mammalian glycans, including hybrid- and complex- types. The glycosylation site N179 is conserved in beta- and gammaherpesviruses and is located near the fusion loops that are proximal to the membrane, potentially indicating that this glycan may play a role in host cell attachment.

**Fig 4:**
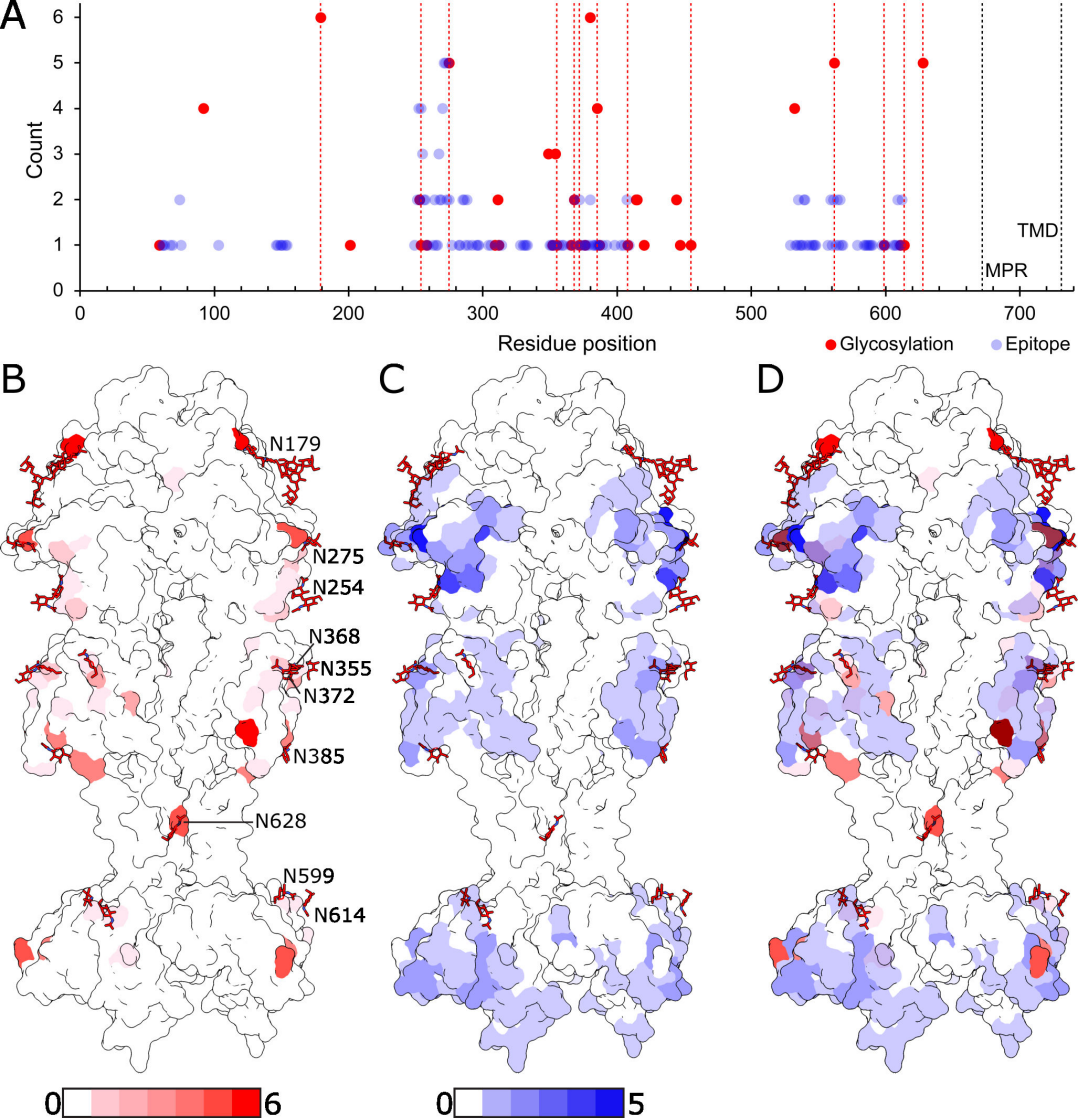
Mapping of human herpesvirus glycosylation and epitope sites on KSHV gB. (**A**) Plot of count of hypothetical glycosylation and experimental epitope sites across all human herpesviruses (HHV1-8, including 6A and 6B) against equivalent KSHV gB residue position. Dashed red lines indicate positions of KSHV gB glycosylation. Red points indicate total instances of glycosylation (KSHV inclusive) and blue points indicate total instances of antibody contact (<5 Å distance) at corresponding KSHV gB residue position. The epitopes used in the plot are listed in Table S2. (**B**) Mapping of glycosylation instances on surface render of KSHV gB atomic model. (**C**) Mapping of epitopes on surface render of KSHV gB atomic model. (**D**) Merge of panels B and C.

Based on sequence alignment, the glycosylation sites on KSHV gB are either unique to KSHV or only partially conserved among herpesviruses (Fig. 3A; Fig. S6 and S7). For example, N368 is conserved only in EBV, the other gammaherpesvirus. N254 and N275 are conserved in betaherpesvirus HCMV, but not in EBV. N628 is the only glycosylation site that is conserved between alpha- and gammaherpesviruses. This unique glycosylation pattern of KSHV gB would cause steric conflicts with experimentally determined epitope sites of other human herpesviruses (15, 20, 26, 32, 34–36) (Fig. 4; Table S2), which suggests that the antibodies against KSHV gB bind to different sites from those against other herpesviruses. The regions occluded from antibody binding would be larger on a fully glycosylated fusogen (Fig. S7). Taken together, the KSHV gB structure reveals by far the most extensively glycosylated among the herpesvirus gB structures reported to date and informs distinct glycan-shielded surface areas that would influence the antigenicity of the gB.

### Structural features of KSHV gB and comparison with other herpesvirus gB

**Fig 5:**
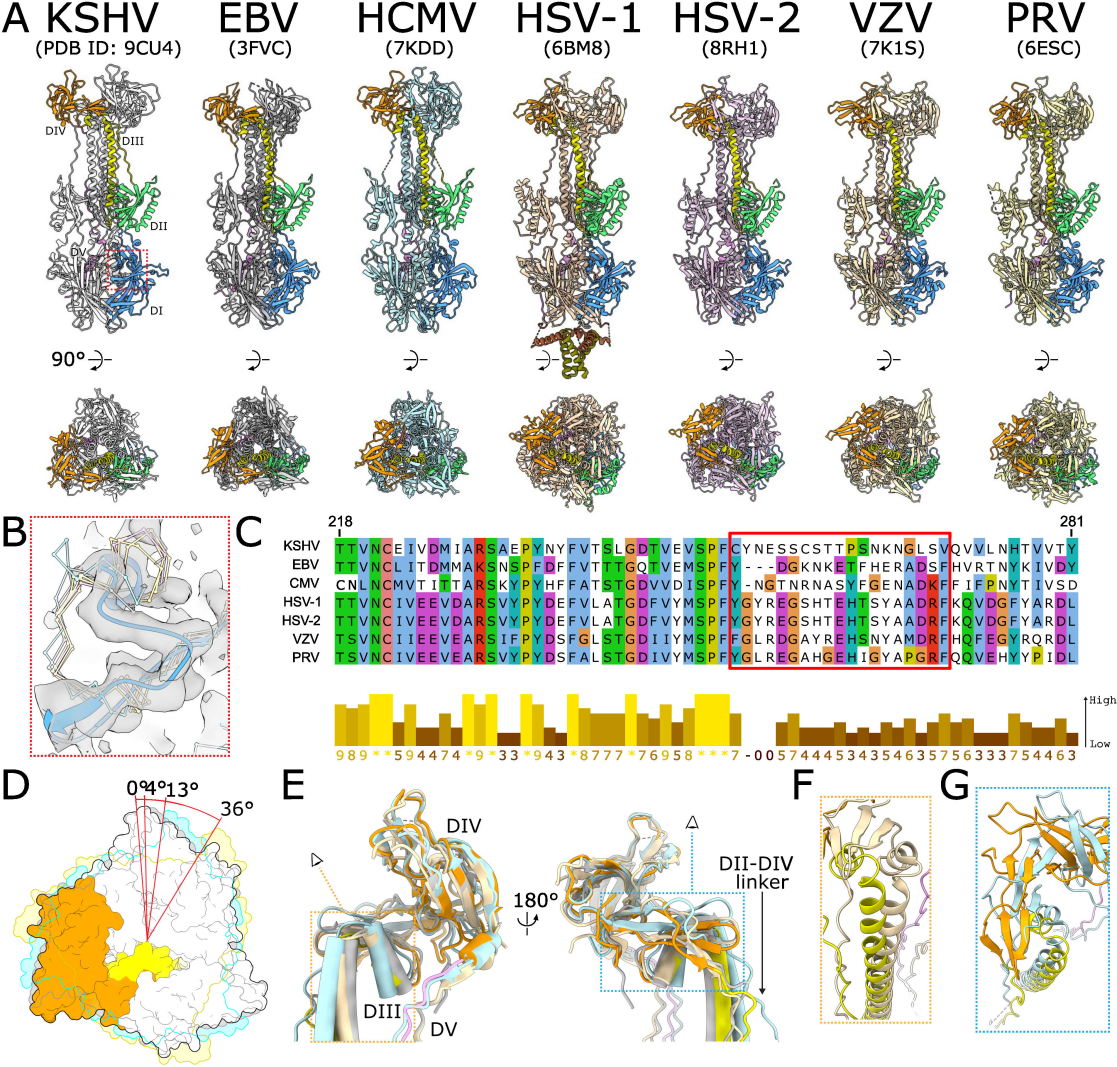
Structural comparison of herpesvirus gB structures. (**A**) Array of gB trimer atomic models from different herpesvirus species. One protomer from each model is colored by domain, while the other two are colored uniquely for each species. Atomic models are aligned by DI down the C3 axis. (**B**) Zoomed in comparison of atomic models of location indicated in (**A**) superimposed with KSHV gB cryoEM map. KSHV gB model is depicted as ribbon colored by domain, and other gB models are depicted as backbone with their representative color. (**C**) Sequence alignment of herpesvirus gB with red box, indicating the region where KSHV gB differs in (**B**). Secondary structures in KSHV gB are indicated above the corresponding residues. (**D**) Overlay of view down the C3 axis from DIV end for KSHV (white), EBV (gray), HCMV (cyan), and VZV (yellow). DIII and DIV for one KSHV gB protomer are colored by domain. Degrees of clockwise rotation of DIV in other species relative to KSHV are indicated. (**E**) Comparison of gB atomic models (KSHV colored by domain; others in representative color) aligned by DIV to identify differences in trajectory of DII-DIV linker, DIII, and DV. (**F**) Comparison of KSHV and VZV gB DIII from perspective indicated in (**E**). (**G**) Comparison of KSHV and HCMV gB DIII-DIV interface helix and β-turn from perspective indicated in (**E**).

We compared our KSHV gB atomic model with prior solved atomic models of other herpesvirus gB (13–20, 26, 32, 34–39). The protomer fold is highly conserved across all herpesviruses (Fig. 5A). In general, the folds of each domain are nearly identical between herpesvirus species, but we observe that a loop in DI of KSHV gB at residues 252–270 deviates greatly from all currently known gB structures (Fig. 5A and B). In KSHV, this loop runs along the exterior of the trimer rather than being tucked in towards the interprotomer interface as in gB of all other species (Fig. 5B). Our cryoEM map of KSHV gB possesses density for this path and lacks density for the conformation followed by other gB (Fig. 5B). Sequence alignment shows that this loop has low conservation between KSHV and other herpesviruses, including its gammaherpesvirus relative EBV (Fig. 5C). This divergent evolution of the KSHV gB DI loop could impact the activity and immunogenicity of this fusogen.

Although the domains within gB are similar between herpesviruses, the orientation of these domains relative to each other differ. Looking down the C3 axis from DIV when the models are aligned by DI, we observe rotational differences between the domains. In KSHV and EBV, DIV lines up with DI and DII of its clockwise neighbor in an eclipsed conformation (Fig. 5A). In HCMV, the sole betaherpesvirus representative with atomic models of gB, DIV is slightly rotated and DII peeks out from below. Additionally, the DIV crown of KSHV gB shares a similar assembly with those of all other herpesviruses, except for HCMV, whose DIVs are bound closer together. For the alphaherpesvirus, DIV is offset from DI and DII in a staggered conformation. By overlaying the DIV crowns, we quantify the rotation between KSHV and EBV, HCMV, and VZV as ~4°, 13°, and 36°, respectively (Fig. 5D). This rotation of DIV relative to DI and DII is conserved between EM and crystal structures within the same herpesvirus species, and only slight differences are present between members within a subfamily.

To observe the contributors to the rotation of DIV, we aligned the atomic models by DIV (Fig. 5E). While the main fold of DIV is conserved, the adjoining DII-DIV linker, DIII, and DV project from DIV with varying trajectories depending on the subfamily. In gB of alphaherpesviruses, DIII bends as it approaches DIV, which shifts the position of the short helix and initial antiparallel β-sheet composed of the β-turn following the helix and a β-strand from the DII-DIV linker (Fig. 5F). Although the trajectory of DV differs between species, its binding with the DIII coiled coil suggests that this difference is a result of the DIV rotation rather than a cause. For HCMV gB, the β-strand following the short helix of DIII is shorter, which shifts the β-turn such that it does not interact with the DII-DIV linker to form an antiparallel β-sheet (Fig. 5G). This in turn permits DIV to turn further inwards towards the C3 axis compared to with KSHV. DIV transitions from being the core of the prefusion state to a distal crown in the postfusion state (26), and the postfusion rotational differences observed between herpesvirus species may have origins in differences between the prefusion states.

### DIII-DIV interdomain dynamics

**Fig 6:**
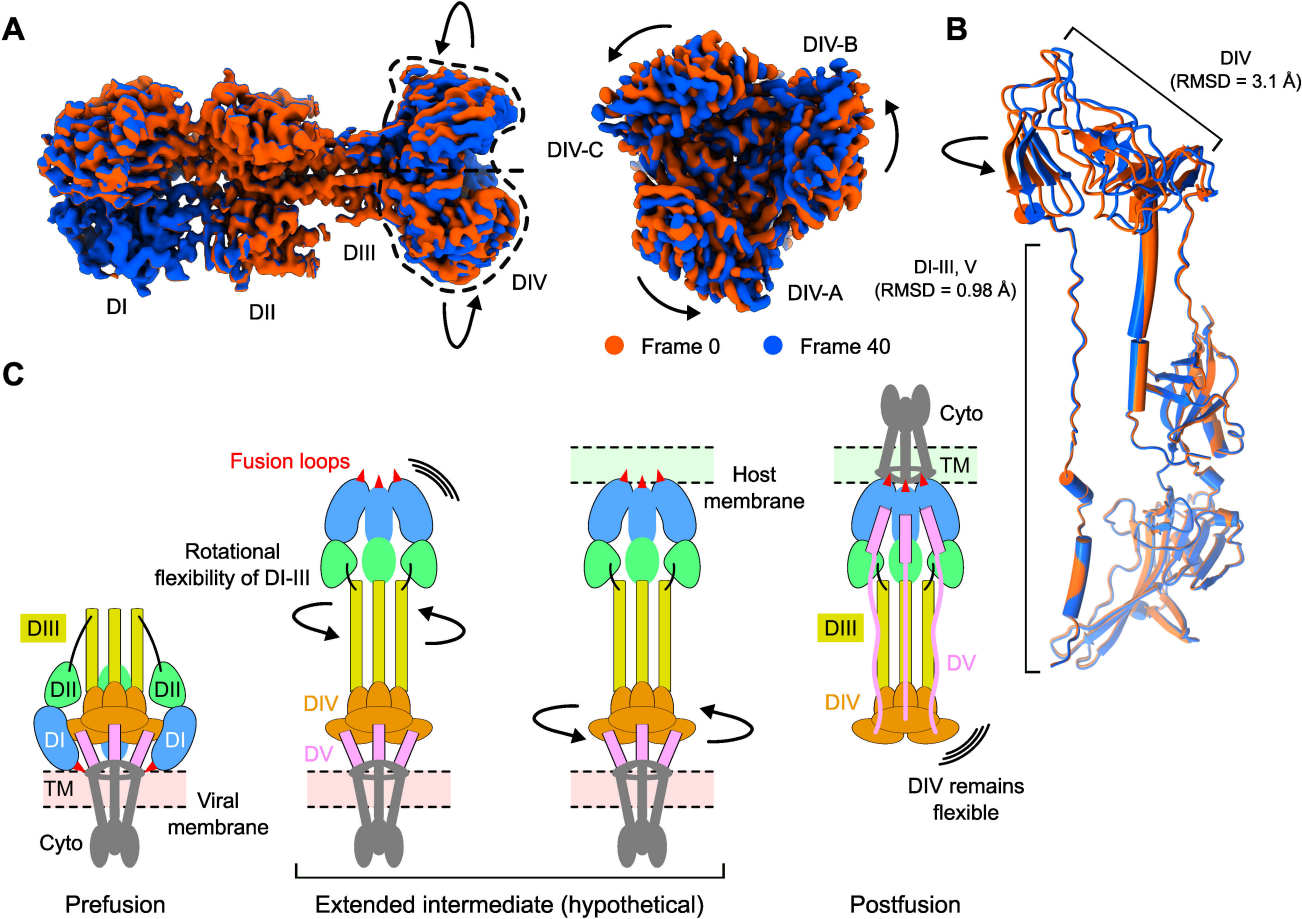
Interdomain dynamics of DIII–DIV. (**A**) Representative frames (first and last) from the 3D flexibility analysis viewed from the side (left) and bottom (right). (**B**) Structural superimposition of the models derived from the first and last frames from the 3D flexibility analysis. RMSD values for the DIV and the remaining ectodomain are indicated. (**C**) Proposed role of the interdomain flexibility present in the gB during the pre- to postfusion conformation change.

Among the five subdomains of the KSHV gB ectodomain, the membrane-distal DIV trimer region showed a lower local resolution, ranging from 3.2 to 4.4 Å, compared to with the rest of the ectodomain, which showed a resolution ranging from 2.6 to 3.4 Å (Fig. S3C). One possible reason for the relatively low resolution in the DIV is the presence of conformational heterogeneity. In addition, when compared with the crystal structure of EBV gB (PDB ID: 3FVC) (14), the largest structural deviations were observed around the DIV region. To understand the conformational landscape of KSHV gB present in our cryoEM data set, we performed 3D flexibility analysis, a generative neural network method that samples continuous heterogeneity within a 3D density map (40). The analysis reveals structural variability around the DIV trimer region with respect to the remaining ectodomain (Fig. 6A). The DIV trimer region exhibits a rotational movement around the three-fold symmetry axis, while the remaining ectodomain (DI-DIII, DV) remains largely static (Fig. 6A; Movie S1). This rotation does not induce major structural alterations within the DIV protomer or its trimeric assembly, suggesting that the domain junctions act as molecular hinges facilitating this movement. Specifically, the flexible hinge regions are located at the DIII-DIV and DIV-DV junctions (amino acids I500-V514 and S619-T623, respectively). Atomic models derived from the first and last frames of the 3D flexibility refinement show an R.M.S.D. of 3.1 Å around the DIV and 0.98 Å across domains I, II, III, and V (Fig. 6B).

During the transformation from prefusion to postfusion state, herpesvirus class III fusogens undergo global conformational transitions, where the DIII central helices invert relative to the DI-DII tandem domains (Fig. 6C). During this transition, gB hypothetically adopts an extended intermediate state in which DI-DII regions are released from the membrane-associated MPR, positioning the hydrophobic fusion loops at the tip of DI for attachment to the host membrane. The DIII-DIV interdomain flexibility potentially provides additional room for the DI fusion loops to fine-tune their positions for insertion into an optimal site on the host membrane surface. This flexibility may also facilitate the transition from the intermediate state to the postfusion form, wherein the DV refolds and folds back along the entire ectodomain, driving the membrane-anchored MPR/TM regions to merge the viral and host membranes. Due to the flexibility needed for these processes, the DIV region remains flexible in the postfusion form even after membrane fusion has completed (Fig. 6C).

## DISCUSSION

Here, we determined the structure of the KSHV gB ectodomain in the postfusion conformation. Despite the DIII hinge mutation, the recombinant ectodomain conforms to the canonical postfusion global fold (Fig. 2). However, KSHV gB possesses a glycosylation pattern distinct from those of other herpesviruses and includes an extended glycan near the fusion loops (Fig. 3). The surface of the herpesvirus gB is predominantly covered by host-derived glycans, a common feature of diverse viral membrane fusion proteins (41). Because herpesvirus gB is a crucial target in vaccine development efforts, understanding the glycosylation of viral fusogens is vital for elucidating the fundamental mechanisms of viral cell entry and for informing vaccine design strategies (42–45).

The KSHV gB structure in the present study displays 30 N-linked glycans in each trimer. These sites are only partially conserved across herpesviruses, with half being specific to KSHV. Each protomer possesses three glycans in DI, four in DII, two in DIV, and one in DV, with no glycan present in DIII.

Notably, the glycan at N179, located on the membrane-proximal side of the DI, showed an extended sugar chain structure as the larger part of the glycan is stabilized through the interactions with the surface residues of the gB. This is the most extensive form of glycan observed in any herpesvirus gB structure to date, as the long glycans are typically too flexible to be resolved by X-ray crystallography or cryoEM. High-mannose type glycan could be modeled most plausibly into the cryoEM density at this site, consistent with the previous study showing that KSHV gB is rich in oligomannose (46). Besides N-linked glycans, O-linked glycans are expected in the two unresolved regions of the ectodomain based on the mass spectrometry-based glycoproteomics in herpesvirus gB homologs (30, 47): immediately after the signal peptide at the N-terminal end and in the DII-DIII linker preceding the furin cleavage site, both regions being rich in proline, serine, and threonine.

The extensive glycosylation on KSHV gB leaves a limited protein surface available for antibody binding. Epitopes previously identified in herpesvirus gB homologs may not be compatible with KSHV gB (Fig. 4). For example, the epitope region in DII observed in EBV gB would be inaccessible for antibodies in KSHV gB due to a KSHV-specific glycan at N372 (32). On the other hand, epitopes in DIV of EBV gB and DI of HCMV gB could be antigenic in KSHV gB as well (15, 32). A fully glycosylated fusogen is expected to occlude a larger surface and further limit conserved epitopes (Fig. S7A). Precise mapping of antigenic surfaces on KSHV gB, however, awaits further epitope mapping study.

The DIII hinge mutation in our KSHV gB construct was added based on a previous cryoET study of an HSV-1 gB hinge mutant present on vesicles in the prefusion state (22). The prefusion state induced by this hinge mutation has been recapitulated in VZV gB expressed on vesicles (23). Notably, the HSV-1 gB hinge mutant ectodomain crystallized in the postfusion conformation, suggesting that the membrane is important for maintaining the prefusion state (22). Although we determined the structure of our KSHV gB hinge mutant ectodomain using cryoEM, which is not subject to conformational changes induced by crystal packing, we also observed the postfusion conformation, which further supports the critical function of the membrane for stabilizing the prefusion state. Indeed, even crosslinked HCMV gB with fusion inhibitor from the native virion has been found to mostly occupy the postfusion state after purification from the membrane (26). Nonetheless, a full-length gB structure spanning from prefusion to postfusion in the context of the membrane would elucidate how herpesviruses undergo membrane fusion.

Like our mutant KSHV virions, mature virions have also been observed for VZV virions containing the gB E526P or V528P DIII hinge mutation attached to the cell surface after budding (23). The recovery and observation of mature virions suggest that the virus can successfully undergo secondary envelopment and egress across the host membrane. Completion of these steps in the viral life cycle implies that fusion can still occur despite the hinge mutation. However, gB knock out HSV-1 viruses are still able to be enveloped and secreted (7), indicating that the secondary envelopment and budding steps can still occur independent of gB. Indeed, the VZV gB V528P mutant virions in particular failed to replicate due to disrupted gB biosynthesis (23). Unlike this VZV gB mutant, the KSHV gB D470P is expressed comparably to the wild-type in transfected cells (Fig. 1). Our structure of KSHV gB D470P mutant still conforms to the canonical postfusion gB ectodomain (Fig. 2D and 5A). Unlike gB from HSV-1 and VZV expressed on extracellular vesicles, a hinge mutation D470P in the KSHV gB soluble ectodomain did not prevent the straightening of the long DIII helix, a typical feature for the postfusion form. This observation also suggests that the disruption in gB biosynthesis is not due to misfolding of the protein. But to truly understand the fundamental cause of the disruption of virion incorporation by the D470P mutant, the structure of gB and its interacting partners would need to be determined in the cellular context. Moreover, due to the lack of gB on the mutant KSHV, this system cannot be used to enrich the presence of prefusion gB for the identification of prefusion gB localization and interactions. Because of the low presence of prefusion gB on WT KSHV virions and low contrast of proteins within cells, advances in structural biology methods are required to fully characterize the mechanism of herpesvirus fusion in action, from prefusion to postfusion and the intermediates in between.

## MATERIALS AND METHODS

### Plasmids

ORF8 (gB) from the KSHV genome (human herpesvirus 8 strain JSC-1 clone BAC16, complete genome; GenBank accession no. GQ994935.1) was cloned into pCAG expression vector under control of CMV enhancer and chicken β-actin promoter. For purification and isolation, a FLAG tag was placed between residues A23 and A24 after the endogenous signal sequence. A truncated version of gB, consisting of residues 1–687, was generated by removing the C-terminal membrane proximal region (MPR) and the sequence beyond it. Based on the prefusion HSV-1 gB structure study (22), a substitution of D470P in the corresponding hinge region was introduced into the truncated gB with the intention to lock the prefusion conformation. The protein expression plasmids of full-length gB with mutations D470P, G471P, or I472P of ORF8 were constructed by oligo mutagenesis through HiFi assembly approach (New England Biolabs E2621L).

### Generation of KSHV with D470P or G471P substitution in the gB coding region

Recombinant KSHV mutant viruses were separately generated with en passant mutagenesis to substitute D470 or G471 of ORF8 with proline and add the FLAG tag at the C-terminus using the BAC16 genetic system in GS1783 *Escherichia coli* in accordance with previously described protocols (48). BAC DNA containing the KSHV D470P genome was analyzed by restriction enzyme digestion and Sanger sequencing to ensure no gross rearrangements occurred and was transfected into 293T cells using Lipofectamine (Thermo Fisher Scientific 11668027). 293T cells stably harboring the viral genome were selected with 100 mg hygromycin. The 293T cells supplemented with wild type gB by transfection were cocultured with iSLK cells in the presence of 20 ng/ml 12-O-tetradecanoyl-phorbol-13-acetate (Fisher Scientific AAJ63916MCR) and 1 mM sodium butyrate (Fisher Scientific AC263191000) to induce virion production and subsequent infection of iSLK cells. Infected iSLK cells were gradually selected with hygromycin from 100 μg/ml to 1.2 mg/mL to establish iSLK-D470P or iSLK-G417P cell line. Expression of gB protein was examined by western blotting of lysates from iSLK-D470P or iSLK-G471P cells that were treated with 1 mM sodium butyrate (NaB) and 5 µg/mL tetracycline to reactivate KSHV and induce lytic replication. To obtain KSHV virions for structural studies, the supernatant was harvested at day 5 after iSLK-D470P or iSLK-G471P cells were treated with 1 mM NaB and 5 µg/ml doxycycline, when >90% of cells exhibited cytopathic effects.

### Western blotting

Western blotting was performed to detect expression of mutant gB proteins either from transfection of expression plasmids in 293T cells or from lytic replication of KSHV in iSLK-D470P or iSLK-G471P cells. For plasmid gene expression, 4 µg of plasmids were transfected into 293T cells in six-well plates (Lipofectamine 2000), and the cell lysates in 400 µL RIPA buffer were collected 2 days post transfection. For viral gene expression, iSLK-D470P or iSLK-G471P cells were treated with NaB and tetracycline, and the cell lysates were collected in RIPA buffer 2 days after reactivation. The supernatant medium was also collected to examine the release of gB protein from infected cells. Equal volumes/amounts of each sample were loaded for western blots and then probed with anti-FLAG antibody.

### Protein expression and purification

The proteins were transiently expressed in ExpiCHO™ (Chinese hamster ovary) cells (Thermo Fisher Scientific). The cell culture and DNA plasmid transfection were performed by following the manufacturer’s instructions. Briefly, the ExpiCHO cells were cultured at an initial density of 0.2 million cells/mL and grown to a density of 6 million cells/mL and transfected with plasmid DNA incubated with ExpiFectamine™ CHO Transfection Kit (Gibco Catalog number: A29131). Typically, 200 mL of transfected cells were harvested 72 h post-transfection. The cells were resuspended with a lysis buffer (20 mM Tris-HCl [pH 8.0] and 150 mM NaCl) supplemented with 2 mM PMSF, lysed by homogenization, and cellular debris was removed by centrifugation. The recombinant gB proteins were purified using EZview Red ANTI-FLAG M2 Affinity Gel (Millipore Sigma) and competitively eluted by 250 µg/mL FLAG peptides (GenScript) in the lysis buffer. The eluted proteins were concentrated to 0.5 mg/mL and underwent buffer exchange to remove residual FLAG peptides. The freshly purified proteins were immediately used for preparing cryoEM grids.

### Negative-stain EM

Approximately 8 µL of 0.02 mg/mL purified gB sample was applied onto glow-discharged ultrathin formvar/carbon supported copper 400-mesh grids (EMS), blotted, and stained with 2.0% uranyl acetate. Negative-stained grids were imaged on a Tecnai F20 transmission electron microscope (FEI) operated at 200 kV.

### CryoEM sample preparation and data acquisition

3 µL aliquots of the purified proteins were applied to glow-discharged Quantifoil R2/1 Cu 300-mesh grids (Quantifoil, Germany). These grids were then blotted for 10 sec at 100% humidity and vitrified in a mixture of liquid ethane and propane (at a 3:7 ratio) cooled by liquid nitrogen using Vitrobot Mark IV (Thermo Fisher Scientific). Vitrified grids were screened in a Tecnai F20 (FEI) transmission electron microscope to optimize the freezing conditions. Subsequent cryoEM data were collected on a Titan Krios G3i (Thermo Fisher Scientific) equipped with a K3 direct electron detector and post-BioQuantum GIF energy filter (Gatan) operated at 300 kV in electron counting mode. Movies were collected at a nominal magnification of 105,000 × in super-resolution mode after binning by a factor of 2, resulting in an effective pixel size of 0.86 Å. A total dose of 50 e^-^/Å^2^ per movie was used with a dose rate of 15–20 e^-^/pix/s. Initial assessment of a small data set showed that KSHV gB has a preferred particle orientation, with its top view predominantly observed in the 2D class averages. To sample more diverse particle orientations, movies were collected with the stage at both zero tilt and a 30 degree tilt (49). In total, 11,911 movies were recorded without stage tilt and 2,722 movies at 30 degrees tilt by automated data acquisition with EPU version 3.5.0.

### CryoEM data processing

The movies were imported into cryoSPARC software package (50) and subjected to patch motion correction and CTF estimation in cryoSPARC. Initially, reference-free manual particle picking in a small subset of data was performed to generate 2D templates for auto-picking and to assess the data quality. Subsequently, 4,041,505 and 1,501,414 particles were picked initially from the untilted and 30 degree tilt data sets, respectively. The particles were extracted, and iterative rounds of 2D classification were performed. Then, 215,660 particles from the 2D class averages with clear secondary structure features were selected from the untilted data set and used for the 3D *ab initio* reconstruction to generate two initial volumes. The particles from the last round of the 2D classification of the untilted data set were reselected to salvage initially missed good particles, which resulted in 249,493 particles. These particles from the untilted data set were combined with 58,924 particles from the 2D classification of the 30 degree tilt data set and used in the following heterogeneous refinement with two copies of each of the two *ab initio* classes as starting volumes. A single class containing 21.9% of the particles showed a feature of postfusion form of class III fusion proteins. Non-uniform refinement (51) was performed with C3 symmetry to yield the final 3.3 Å resolution map. We did not observe any prefusion-like 2D or 3D classes in these data sets. To investigate the continuous motion around DIV region, a cryoSPARC (v.4.3.1) 3DFlex (40) training model was generated with two latent dimensions, and a rigidity prior (lambda) of 1. 41 frames were generated to visualize the volume series.

### Atomic model building

The crystal structure of postfusion EBV gB (PDB ID: 3FVC) (14) was used as a homology model and fitted into the cryoEM density map using ChimeraX (52). The model was then mutated and manually refined in Coot (53), followed by iterative refinement using ISOLDE (54) and Phenix real-space refinement (55). To build glycan chains, the carbohydrate module in Coot was used with mammalian N-linked glycosylation restrains. Atomic models and maps were visualized in Coot and ChimeraX. There are unknown densities near the DI-DI trimer interface and inside the cavity formed by the trimer (Fig. S4B). These densities did not appear to be part of the proteins or glycans and were left unmodelled. Sequence alignments were performed with Clustal Omega and EMBOSS Needle on the EMBL-EBI server (56) and visualized in Jalview (57) or Linneao (https://github.com/beowulfey/linnaeo).

### KSHV culture and isolation

KSHV virions were prepared as previously described with slight modification from iSLK-KSHV-BAC16 cells, received as a gift from Dr. Jae U. Jung (48, 58). In brief, iSLK-KSHV-BAC16 cells were cultured in Dulbecco’s Modified Eagle Medium (DMEM; Corning 10-017-CV) supplemented with 10% fetal bovine serum (FBS; 35-010-CV) and 100 U/mL of penicillin-streptomycin, 1 mg/mL puromycin (Invivogen ant-pr-1), 250 µg/mL G418 (Invivogen ant-gn-1), and 1,200 mg/mL hygromycin B (Invivogen ant-hg-1). Cells were cultured to 80%-90% confluency in 24 15 cm tissue culture dishes. Then, KSHV lytic replication and virion production were induced by treatment with 1 mM sodium butyrate and 5 µg/mL tetracycline for five days, after which 720 mL of media containing KSHV virions were collected. Infectious media were clarified by centrifugation at 10,000 × g for 20 min at 4°C twice. Virions were then pelleted using an SW28 rotor (Beckman) at 80,000 × g for 1 h at 4°C. The supernatant was discarded, and 50 µL of cold phosphate-buffered saline (PBS) was added to the pellet in each tube. The pellets were allowed to soften for 16 h on ice in a 4°C cold room before gentle resuspension by tapping. About 0.7 mL of combined pellet suspension from all ultracentrifuge tubes were loaded on 15%-50% linear gradient of sucrose made in PBS and spun in SW41 (Beckman) rotor at 80,000 × g for 1 h at 4°C. About 1 mL of the visible virus-containing band was collected into a new tube and mixed with 10 mL of sterile PBS solution in order to dilute sucrose. KSHV gB G471P did not have a visible band and was instead fractionated in 1 mL aliquots based on the band locations of other herpesviruses. The virions were concentrated at 80,000 × g for 1 h at 4°C and 20 µL of PBS was added. After softening and gentle resuspension, the isolated virions were recovered and immediately used for cryoET sample preparation without freezing.

### CryoET sample preparation and image acquisition

Virion samples were screened on an FEI Tecnai TF20 equipped with a 4k × 4k TVIPS F415MP CCD detector or Gatan K2 detector. To prepare EM grids for cryoET imaging, 3 µL of diluted virions were mixed with 10 nm gold fiducials and applied to Quantifoil R2/1 Cu 200 grids and plunge-frozen in liquid ethane-propane mixture using a Vitrobot mark IV (FEI Thermo Fisher Scientific) set to 100% humidity, 4°C, and blot time of 5 s.

Imaging was performed with a Titan Krios G1 (FEI Thermo Fisher Scientific) operated at 300 kV, and movies were recorded using a Gatan K3 detector with an energy filter slit width of 20 eV. Tilt series were collected from −60° to 60° in 3° increments using a dose-symmetric scheme (59) using SerialEM (60) at 64,000 × magnification (0.690 Å/pixel super-resolution) and a target focus of -4 µm with a total of dose of 120 e^-^/Å^2^. Individual tilts were recorded with constant beam intensity in 10 frames.

### Tomogram reconstruction

Each movie stack was drift-corrected using MotionCor2 (61), and defocus values were determined with CTFFIND4 (62). Tilt series alignment via gold fiducials and tomogram reconstructions were performed in IMOD with a binning factor of 4 (63). IsoNet isotropic reconstructions with a binning factor of 8 were used for observation (64).

## Data Availability

The atomic model has been deposited in the PDB with accession code 9CU4. The cryoEM map has been deposited in the EMDB with accession code EMD-45927.

## SUPPLEMENTAL MATERIAL

Supplemental material jvi.01533-24-s0001.docx Figures S1 to S7; Tables S1 and S2.

Movie S1 jvi.01533-24-s0002.mp4 KSHV gB experiences a rotational flexibility around DIV.

ASM does not own the copyrights to Supplemental Material that may be linked to, or accessed through, an article. The authors have granted ASM a non-exclusive, world-wide license to publish the Supplemental Material files. Please contact the corresponding author directly for reuse.
