# Supplementary material for: Structure of the Kaposi’s sarcoma-associated herpesvirus gB in post-fusion conformation
Source: J Virol. 2025 Jan 17;99(2):e01533-24. doi: 10.1128/jvi.01533-24 (PMC11852774; doi:10.1128/jvi.01533-24)
Supplement: Supplemental material — Figures S1 to S7; Tables S1 and S2. [file jvi.01533-24-s0001.docx]

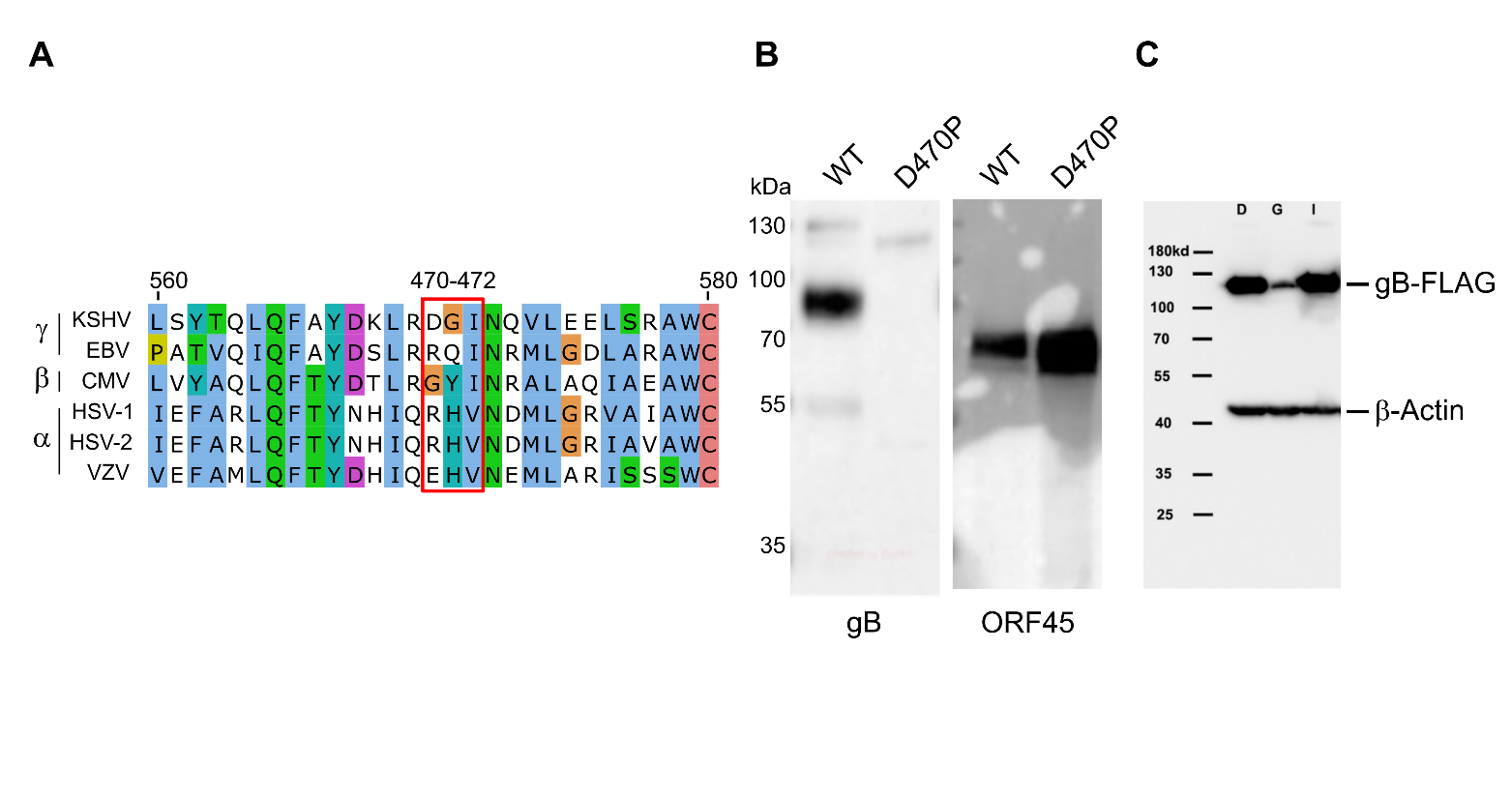


**Fig. S1. Generation of KSHV gB hinge mutants.** (**A**) Sequence alignment of herpesvirus gB near the DIII hinge region. A patch of three-amino acid hinge region is highlighted with a box. (**B**) The mutant gB (D470P) was not incorporated into the KSHV virions. The concentrated virions were prepared from the supernatants of reactivated KSHV-harbored iSLK cells after complete removal of cell debris. Equal amounts of concentrated viral proteins (3 ug) from WT or the D470P virions were loaded for western blots probed with anti-gB (left) or anti-ORF45 antibodies (right). (**C**) Expression of the three hinge mutants of gB in HEK293T cells. D: D470P; G: G471P; I: I472P. The expression of gB is under control of CMV enhancer + Chicken β-actin promoter from a pCAG vector. The membrane was probed with an anti-FLAG antibody.

**
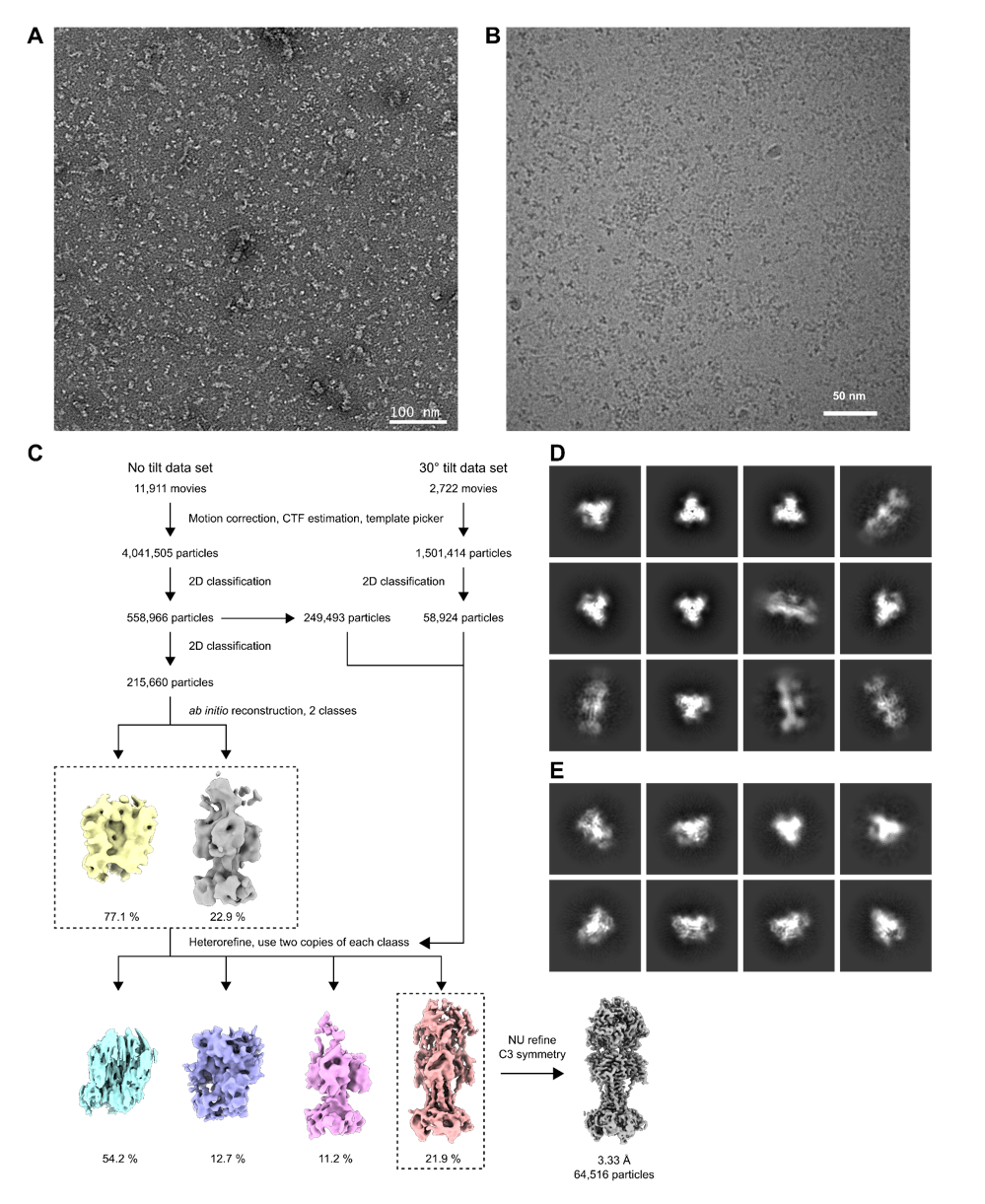
**

**Fig. S2. Workflow and intermediate results of electron microscopy imaging and 3D reconstruction of KSHV gB.** (**A**) Representative negative-stain raw image. (**B**) Representative cryoEM raw image. (**C**) CryoEM image processing workflow. (**D**) Representative 2D class averages of the no-tilt data. (**E**) Representative 2D class averages of the 30-degree tilt data.


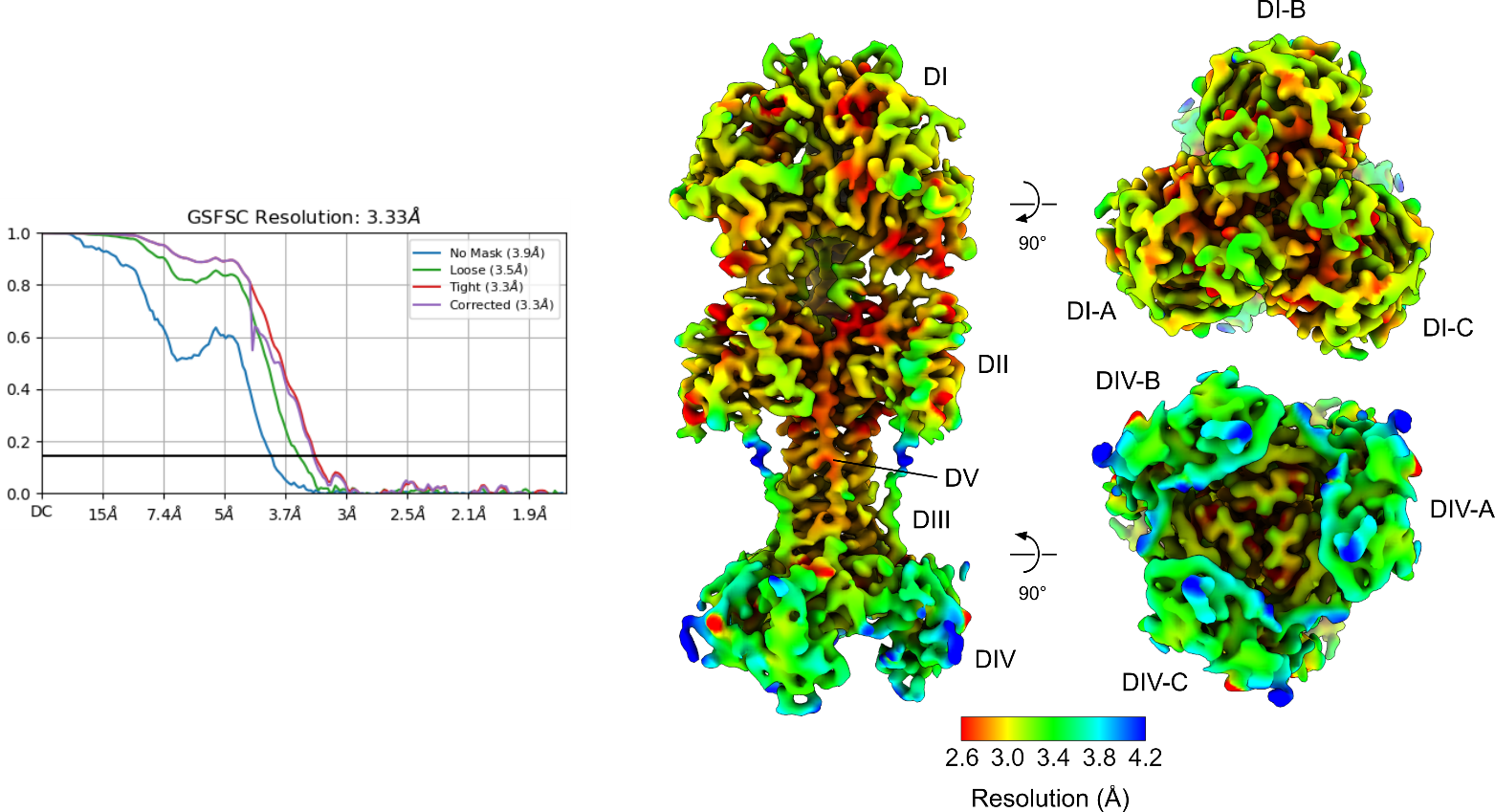


**Fig. S3. Evaluation of the cryoEM reconstruction of KSHV gB.** Global resolution estimation (**A**), angular distribution plot of the particles (**B**), and local resolution evaluation in three orthogonal views (**C**) of KSHV gB. Resolution estimation is based on the gold standard Fourier shell correlation (FSC) coefficient of 0.143 criteria.


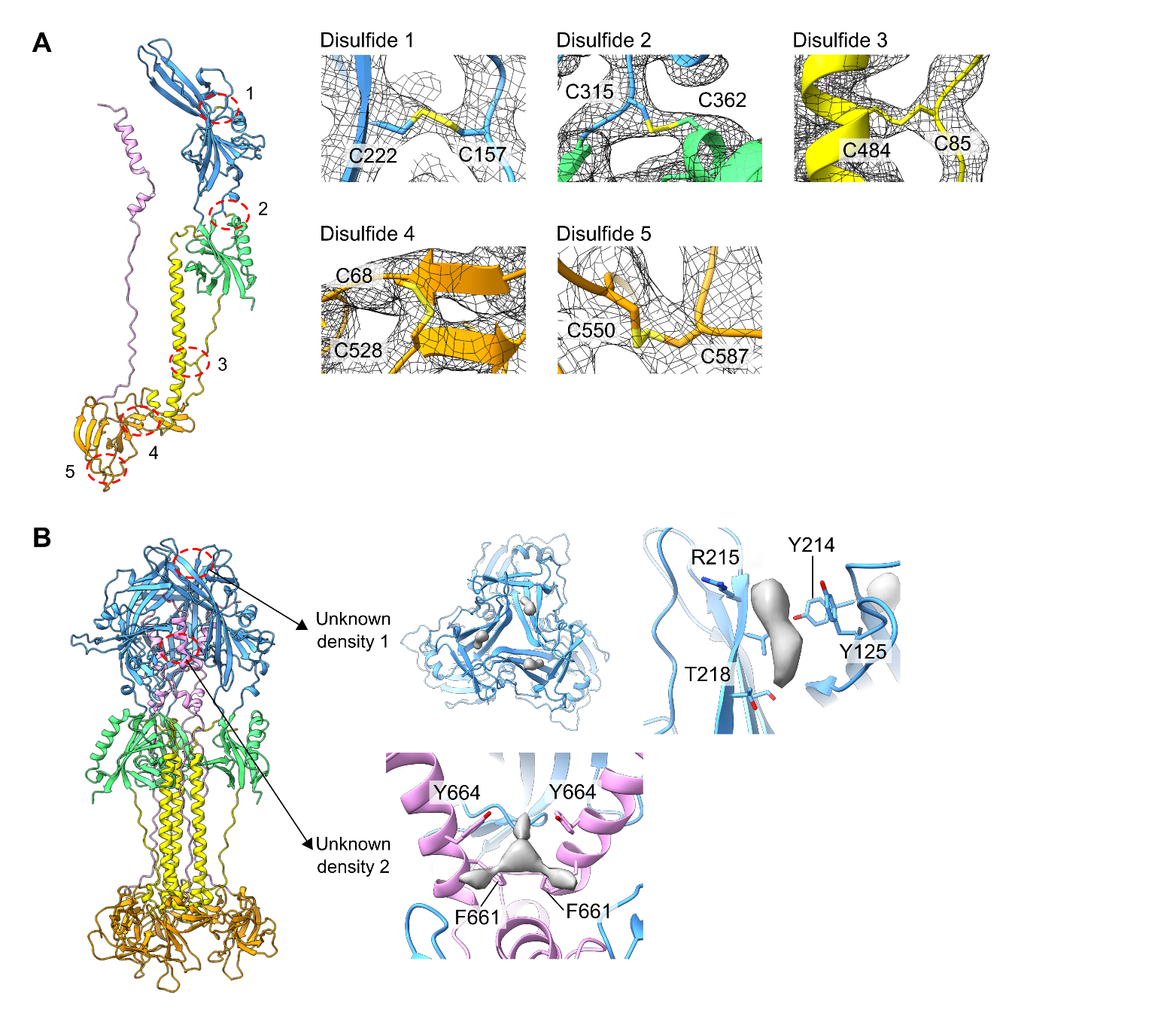


**Fig. S4. Intraprotomer disulfide bonds and unidentified density observed in KSHV gB** (**A**) CryoEM densities of the five conserved disulfide bonds shown as mesh surface. (**B**) Two unassigned densities near the DI-DI trimer interface and inside the trimer cavity near DV.


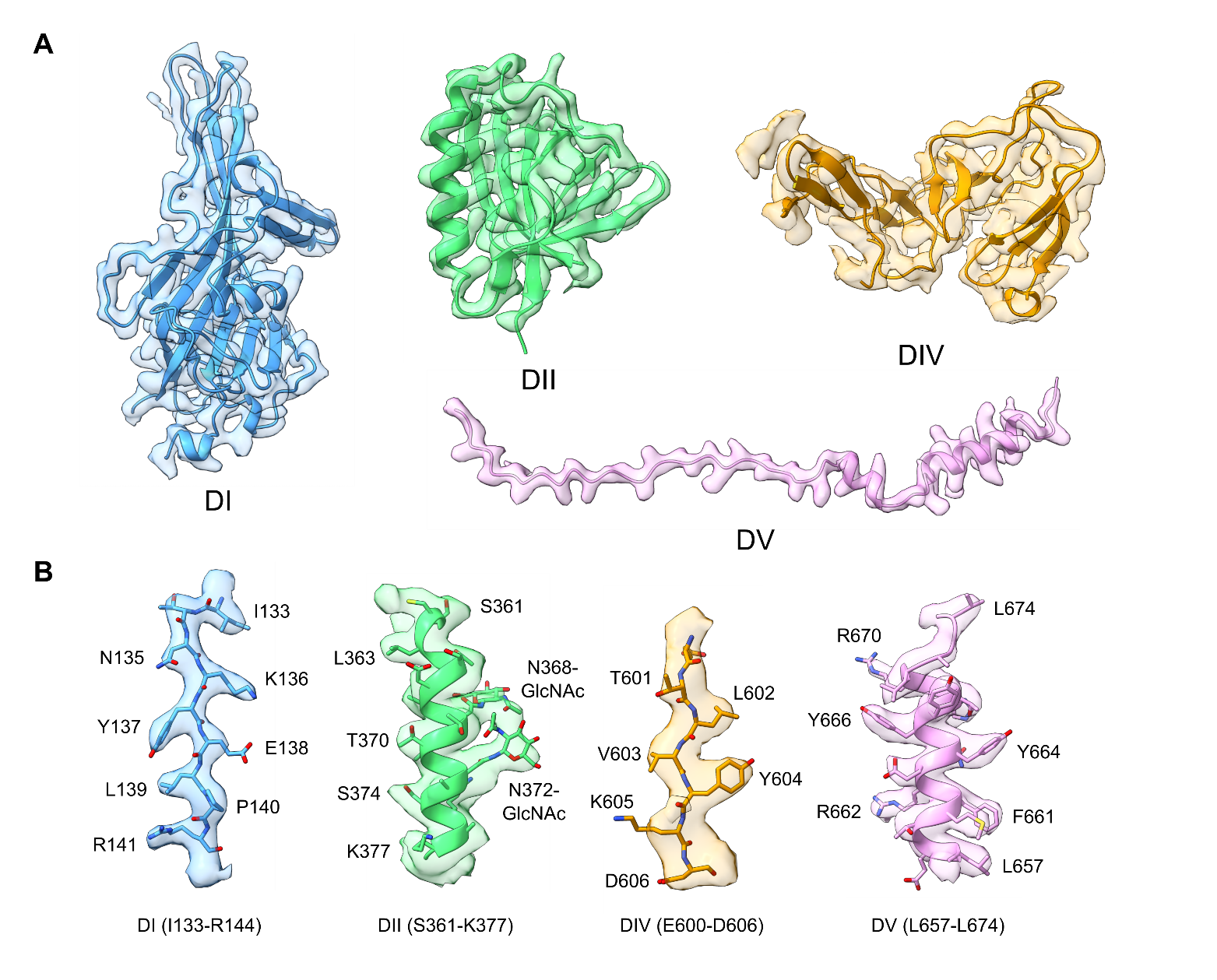


**Fig. S5. Regions of the cryoEM density maps superposed with the atomic model of KSHV gB.** (**A**) Segmented cryoEM densities of DI, DII, DIV, and DV of KSHV gB ectodomain shown as semitransparent surfaces superposed with the corresponding atomic models in ribbons. (**B**) Segmented cryoEM densities of representative local regions of DI, DII, DIV, and DV. The atomic models of amino acid side chains are shown in sticks.


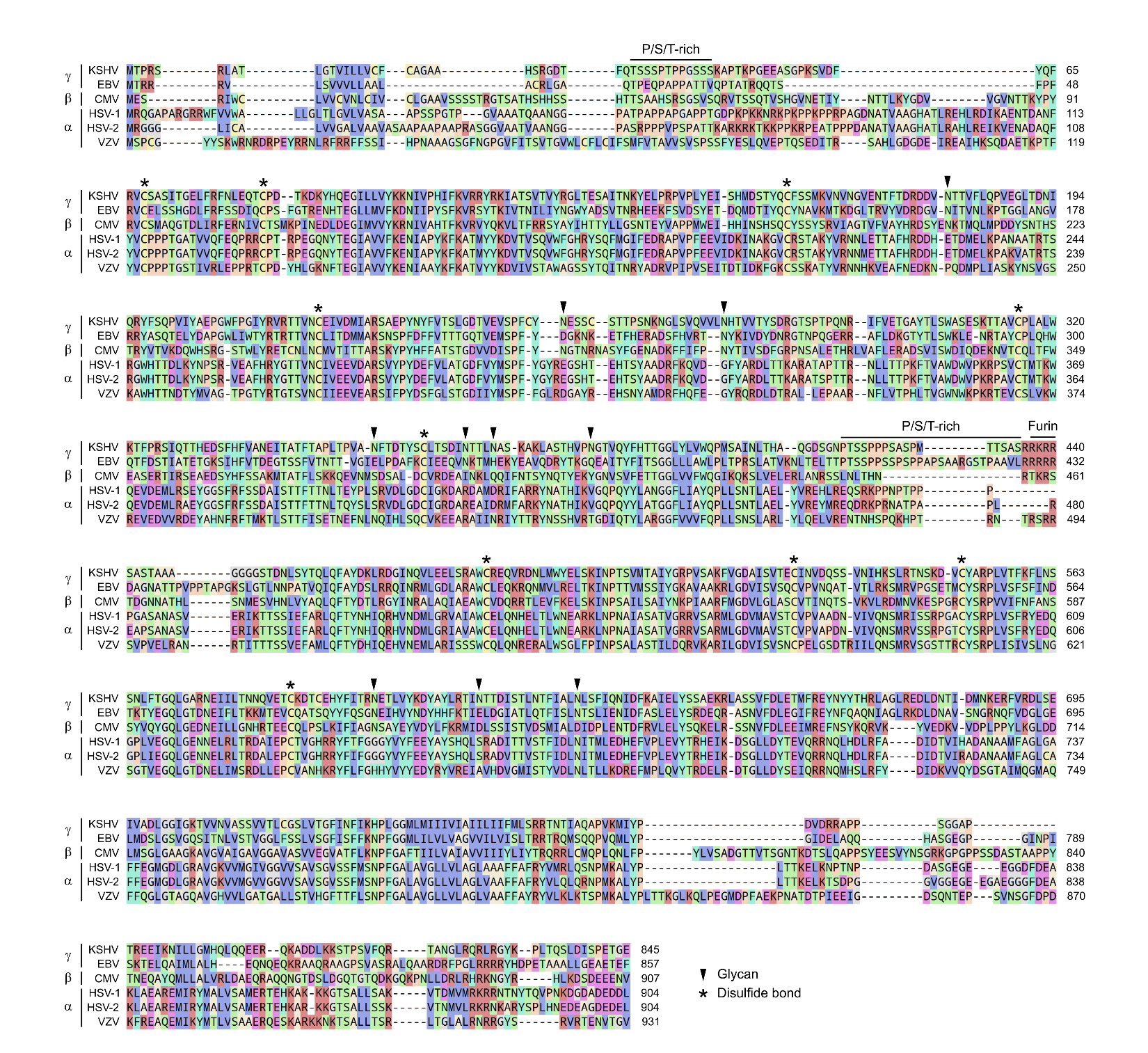


**Fig. S6. Sequence alignment of herpesvirus gB.** The observed glycosylation sites and conserved cysteines that form disulfide bonds are highlighted with triangles and asterisks, respectively.


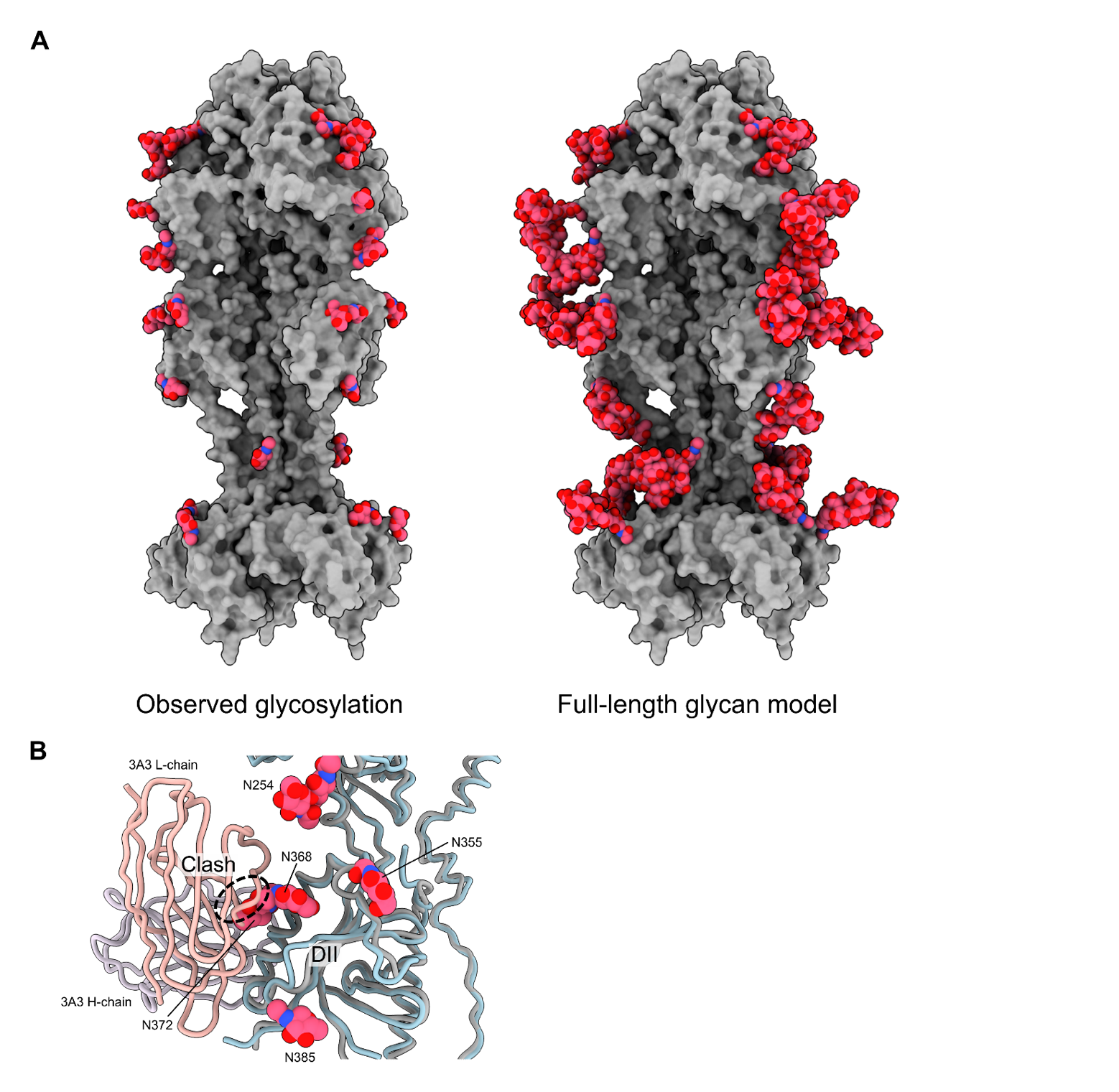


**Fig. S7. Insights into the glycosylation of KSHV gB.** (**A**) Observed glycosylation (left) and fully-glycosylated model (right) of KSHV gB. High-mannose type glycans (Asn-N-GlcNAc2-Man8) were modeled based on the observed glycans in the cryoEM structure. (**B**) Close-up view of the DII region of KSHV gB (tubes in dark grey) overlayed with EBV gB (pale cyan) bound to neutralizing antibody 3A3 (PDB ID: 7FBI). The glycan at N372 would clash with an antibody targeting the equivalent epitope in KSHV gB.

**Supplementary Table 1. Cryo-EM data collection, refinement, and validation statistics**

|  | KSHV gB  postfusion  (PDB: 9CU4)  (EMDB: EMD-45927) |
| --- | --- |
| **Data collection** |  |
| Magnification | 105,000 |
| Voltage (kV) | 300 |
| Electron exposure (e^-^/Å^2^) | 50 |
| Defocus range (μm) | -0.8 to -2.8 |
| Pixel size (Å) | 0.86 |
| Symmetry imposed | C3 |
| Initial particle images | 4,041,505 |
| Final particle images | 64,516 |
| Map resolution (Å) | 3.3 |
| FSC threshold | 0.143 |
| Map resolution range (Å) | 2.6 to 4.4 |
| **Refinement** |  |
| Initial model used (PDB) | 3FVC |
| Model resolution (Å) | 3.6 |
| FSC threshold | 0.5 |
| Map sharpening B factor (Å^2^) | -46.0 |
| No. non-hydrogen atoms | 4896 |
| Protein residues | 585 |
| Ligands | 18 |
| *B*-factors |  |
| Protein | 70.95 |
| Ligand | 76.40 |
| R.m.s. deviations |  |
| Bond lengths (Å) | 0.013 |
| Bond angles (°) | 1.822 |
| Validation |  |
| MolProbity score | 1.25 |
| Clash score | 2.08 |
| Poor rotamers (%) | 0.19 |
| Ramachandran plot |  |
| Favored (%) | 96.04 |
| Allowed (%) | 3.96 |
| Disallowed (%) | 0.00 |

**Supplementary Table 2. List of known epitopes in herpesvirus gB and their equivalent residues in KSHV gB**

| HCMV_1G2  (PDB: 5C6T) | | HCMV_SM5-1  (PDB: 7KDD) | | HSV-1_BMPC-23  (PDB: 7UHZ) | | HSV-1_D48  (PDB: 8KFA) | | HSV-1_HDIT101  (PDB: 8RGZ) | |
| --- | --- | --- | --- | --- | --- | --- | --- | --- | --- |
| Current epitope | Equivalent residue in  KSHV | Current epitope | Equivalent residue in  KSHV | Current epitope | Equivalent residue in  KSHV | Current epitope | Equivalent residue in  KSHV | Current epitope | Equivalent residue in  KSHV |
| T100 | G74 | T100 | G74 | S586 | S541 | R377 | Q328 | G301 | Y252 |
| D101 | E75 | R131 | K103 | R588 | R543 | R418 | N368 | Y302 | N253 |
| Y280 | C252 | E359 | T330 | S590 | N/A | D422 | N372 | R303 | E254 |
| G282 | N254 | A360 | H331 | S591 | N545 | F425 | K375 | E304 | S255 |
| T283 | E255 | E361 | E332 | R592 | S546 | N430 | S380 | G305 | S256 |
| R285 | S257 | D362 | D333 | P593 | K547 | H433 | H382 | S306 | C257 |
| N286 | C258 | K379 | L350 | G594 | D548 | I434 | V383 | H308 | S259 |
| F290 | S263 | Q380 | T351 | I630 | V584 | K435 | P384 | A316 | G267 |
| G291 | N264 | E381 | P352 | E631 | E585 | V436 | N385 | F319 | V270 |
| E292 | K265 | V382 | V353 | P632 | T586 | G437 | G386 | K320 | Q271 |
| N293 | N266 | N383 | A354 | C633 | C587 | Q438 | T387 | Q321 | V272 |
| A294 | G267 | D386 | T357 | T634 | K588 | Q440 | Q389 | V322 | V273 |
| F297 | V270 | A388 | T359 | V635 | D589 | P454 | P403 | D323 | L274 |
| F298 | Q271 | L389 | Y360 | G636 | T590 | L456 | S405 | G324 | N275 |
| I299 | V272 | E422 | H392 | E652 | D606 | N458 | I407 | Y326 | T277 |
| P301 | L274 | V428 | Y398 | Y653 | Y607 |  |  | P339 | P290 |
| L321 | N292 |  |  | A654 | A608 |  |  | T340 | T291 |
|  |  |  |  |  |  |  |  | T341 | T292 |
|  |  |  |  |  |  |  |  | W356 | S307 |
|  |  |  |  |  |  |  |  | P358 | S309 |

| HSV-1_HDIT102  (PDB: 8RH0) | | HSV-1_HSV010-13  (PDB: 7UI0) | | HSV-2_HDIT101  (PDB: 8RH1) | | HSV-2_HDIT102  (PDB: 8RH2) | | VZV_93k  (PDB: 6VN1) | |
| --- | --- | --- | --- | --- | --- | --- | --- | --- | --- |
| Current epitope | Equivalent residue in  KSHV | Current epitope | Equivalent residue in  KSHV | Current epitope | Equivalent residue in  KSHV | Current epitope | Equivalent residue in  KSHV | Current epitope | Equivalent residue in  KSHV |
| D199 | S149 | V115 | V67 | S293 | S249 | E190 | Y146 | T115 | D61 |
| A203 | S153 | P117 | S69 | F295 | F251 | E191 | E147 | K116 | P62 |
| K204 | T154 | V574 | I529 | Y296 | C252 | K195 | H150 | P117 | Y63 |
| Y303 | N254 | R605 | K559 | G297 | Y253 | I196 | M151 | S589 | Q533 |
| R304 | E255 | Y606 | F560 | Y298 | N254 | F295 | F251 | D590 | S534 |
| K320 | Q271 | E607 | L561 | E300 | S256 | Y296 | C252 | R592 | S535 |
| Q321 | V272 | Q609 | S563 | S308 | N264 | D312 | L268 | I593 | V536 |
| V322 | V273 | G610 | S564 | A311 | G267 | R313 | S269 | I594 | N537 |
| D323 | L274 | P611 | N565 | D312 | L268 | F314 | V270 | L595 | I538 |
| G324 | N275 | L612 | L566 | R313 | S269 | K315 | C271 | Q596 | H539 |
| Y326 | T277 | V613 | F567 | F314 | V270 | Q316 | V272 | N597 | K540 |
| R335 | T286 | E614 | T568 | K315 | C271 | D318 | L274 | S615 | T557 |
| T337 | P288 | L625 | L579 | Q316 | V272 | T327 | D283 | V617 | K559 |
|  |  |  |  | D318 | L274 | A329 | G285 | L619 | L561 |
|  |  |  |  | A329 | G285 |  |  | F655 | T597 |
|  |  |  |  | R330 | T286 |  |  | H657 | N599 |
|  |  |  |  | A331 | S287 |  |  | H658 | E600 |
|  |  |  |  | T332 | P288 |  |  | Y667 | Y609 |
|  |  |  |  | S333 | T289 |  |  | E670 | T612 |
|  |  |  |  | W348 | W304 |  |  |  |  |
|  |  |  |  | D350 | A306 |  |  |  |  |
